# Recent evolution of the NF-κB and inflammasome regulating protein POP2 in primates

**DOI:** 10.1186/1471-2148-11-56

**Published:** 2011-03-01

**Authors:** Maninjay K Atianand, Travis Fuchs, Jonathan A Harton

**Affiliations:** 1 Center for Immunology and Microbial Disease, Albany Medical College, Albany, New York, 12208, USA; 2 Department of Biological Sciences, University at Albany, Albany, New York, 12222, USA

## Abstract

**Background:**

Pyrin-only protein 2 (POP2) is a small human protein comprised solely of a pyrin domain that inhibits NF-κB p65/RelA and blocks the formation of functional IL-1β processing inflammasomes. Pyrin proteins are abundant in mammals and several, like POP2, have been linked to activation or regulation of inflammatory processes. Because *POP2 *knockout mice would help probe the biological role of inflammatory regulation, we thus considered whether *POP2 *is common in the mammalian lineage.

**Results:**

BLAST searches revealed that *POP2 *is absent from the available genomes of not only mice and rats, but those of other domestic mammals and New World monkeys as well. *POP2 *is however present in the genome of the primate species most closely related to humans including *Pan troglodytes *(chimpanzees), *Macaca mulatta *(rhesus macaques) and others. Interestingly, chimpanzee POP2 is identical to human POP2 (huPOP2) at both the DNA and protein level. Macaque POP2 (mqPOP2), although highly conserved is not identical to the human sequence; however, both functions of the human protein are retained. Further, *POP2 *appears to have arisen in the mammalian genome relatively recently (~25 mya) and likely derived from retrogene insertion of *NLRP2*.

**Conclusion:**

Our findings support the hypothesis that the NLR loci of mammals, encoding proteins involved in innate and adaptive immunity as well as mammalian development, have been subject to recent and strong selective pressures. Since POP2 is capable of regulating signaling events and processes linked to innate immunity and inflammation, its presence in the genomes of hominids and Old World primates further suggests that additional regulation of these signals is important in these species.

## Background

Initiation of innate immune/inflammatory responses by pathogens results in the secretion of cytokines that recruit phagocytes, increase phagocyte microbicidal activity, promotes antigen presentation and the development of adaptive immunity [1]. To initiate these responses, pathogens must be sensed through one or more host pattern recognition receptors (PRR). PRRs include the Toll-like receptor (TLR), RIG-I helicase-like receptor, or nucleotide-binding, leucine repeat (NLR) receptor families. PRR engagement by pathogen-associated molecular patterns activates receptor-mediated signaling via MAPK, STAT, and/or NF-κB (reviewed in [1-3]). Activation of the MAPK and NF-κB pathways cooperate to drive the gene expression of proinflammatory cytokines such as IL-1β, IL-6, IL-8, and TNFα. Secretion of IL-1β and the IL-1β-related cytokine IL-18, requires processing of the respective pro-forms by caspase-1. Activation of caspase-1 occurs in the context of the dynamic multi-protein inflammasome complex through either direct or ASC (apoptotic speck-like protein containing a CARD)-mediated indirect recruitment via NLR proteins [4,5].

While the molecular basis and regulation of NF-κB signal transduction downstream of PRR family members is well-studied [2,6,7], inflammasome function and regulation is poorly understood. Pyrin domain (PYD) and caspase recruitment domain (CARD) homodomain interactions are important for inflammasome formation, suggesting the potential for CARD-only proteins (COPs) and PYD-only proteins (POPs) to act as negative regulators. COPs (e.g. INCA, ICEBERG, and COP) inhibit Caspase-1 activation by preventing Caspase-1 recruitment to the inflammasome complex [8-10]. Two mammalian POPs have also been discovered. POP1 (ASC2) is highly similar to the PYD of ASC (PyCARD), the adaptor molecule that bridges the PYD of NLRPs to the CARD of Caspase-1 to facilitate inflammasome assembly. Although a potential function, POP1 has not yet been shown to inhibit inflammasome formation/activation [11]. POP2 is more similar to NLR PYDs and effectively inhibits inflammasome activation by limiting the interaction of various NLRPs with ASC [12,13]. Importantly, the inflammasomes influenced by POP2 include NLRP1, NLRP3, and NLRP12 which have been linked to specific inflammatory diseases including atopic dermatitis [14]; the cryopyrin-associated periodic syndromes [15,16], and other hereditary periodic fevers [13]. POP1 and POP2 are also capable of inhibiting NF-κB activation, although the mode of inhibition differs [11,12]. Thus POP2 has the potential to function as a dual regulator of innate immune/inflammatory responses by influencing both inflammasome function and PRR signaling via NF-κB.

Here we report that the genomes of mouse, rat, and a number of other domestic mammals with available complete genome sequence data lack *POP2*. While the available genomes of catarrhine primates (comprising both hominids and Old World monkeys) contain *POP2*, those of New World primates (platyrrhine) do not, strongly supporting the recent evolution of *POP2*. Our data also reveals an increasing number of NLRP2-related sequences during mammalian evolution. A functional analysis of macaque POP2 reveals a protein capable of both NF-κB and inflammasome inhibition, demonstrating that these functions likely coincide with the emergence of *POP2 *some time after the divergence of Old World and New World primates approximately 40 mya. The pattern of *POP2 *evolution and the significance of the recent emergence of both POP1 and POP2 as potential regulators of NF-κB signaling and inflammasome function are discussed.

## Results and discussion

### *POP2 *is absent from the genomes of mice, rats, and other domestic mammals

**Figure 1:**
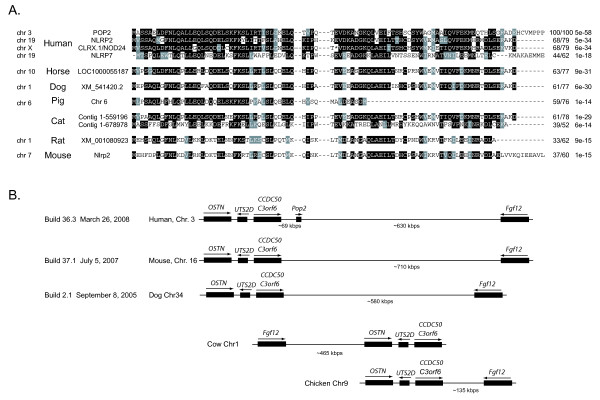
**POP2 is absent from the genomes of mice, rats, and other domestic mammals**. A. Translated BLAST searches of the human, horse, dog, pig, cat, rat, and mouse genome were performed with the POP2 protein sequence. The protein sequence of the PYD for the four POP2 PYD related sequences in human are shown as are those sequences with the highest similarity to POP2 identified from each of the other genomes examined. All the identified sequences represent NLRP genes. Chr, chromosomal location. Shading represents conservation (either identity (black) or functionally similar (gray)) at given positions in >50% of aligned sequences. Percent identity/similarity and expect values are shown at right to the aligned sequences.

To attempt to identify and isolate the mouse equivalent of human POP2, we performed translated BLAST searches of the mouse genome. Curiously, although other PYDs in NLR family members were detected, a sequence with high similarity to POP2 was not. The completed genomes of a variety of other domestic mammals were also examined with similar results. Sequences with the highest homologies in these species were the PYDs of the putative orthologs of *NLRP2 *or *NLRP7*, the genes most closely related to *POP2 *in humans (Figure 1A). Searches were also performed for human POP1, which was also absent in these genomes. As expected, the ASC PYD had the greatest homology to POP1. Since POP2 is encoded by a single exon of 297 base pairs in humans adjacent to the gene for CCDC50 (c3orf6) and approximately 700 kbp upstream of the gene for Fgf12 [12], we examined the corresponding locus in mouse. This locus is present in the mouse genome, has been completely sequenced, and is nearly identical to the human locus in size and gene arrangement with the exception that *POP2 *is absent (Figure 1B). These results suggest that both *POP1 *and *POP2 *may be recent developments in mammalian evolution and raise the possibility that both genes are unique to primates.

### *POP2 *is an evolutionarily young gene present in hominid and Old World primates

**Figure 2:**
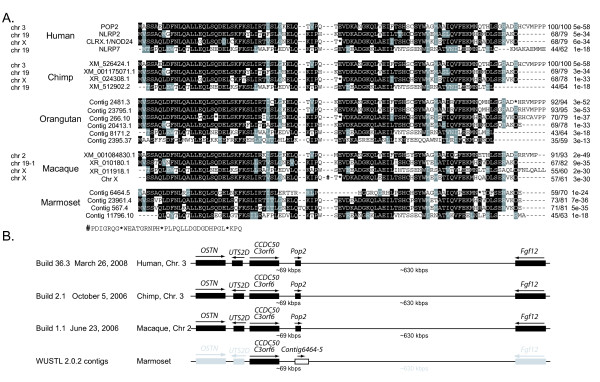
**POP2 is present in hominid and Old World primates**. A. Protein sequences of the PYDs most closely related to that of POP2 from the genome sequences of human, the non-human hominid primates *Pan troglodytes *(chimpanzee) and *Pongo abelii *(orangutan; 6 × coverage), the Old World primate *Macaca mulatta *(Rhesus macaque), and the New World primate *Callithrix jacchus *(marmoset; 6 × coverage). Percent identity/similarity and expect values are shown at right to the aligned sequences. B. Genomic maps of the POP2 loci for genomes with chromosomal map data from the indicated species. Gray indicates presumed gene locations and approximate distances in the marmoset genome. Note: Marmoset contig 6464-5 which corresponds by position with POP2 lacks a start codon and intact open reading frame. Vestiges of the nucleotide binding and leucine-rich repeats analogous to NLRP2/7 are contiguous in the sequence immediate downstream of the PYD-like sequence.

**Figure 3:**
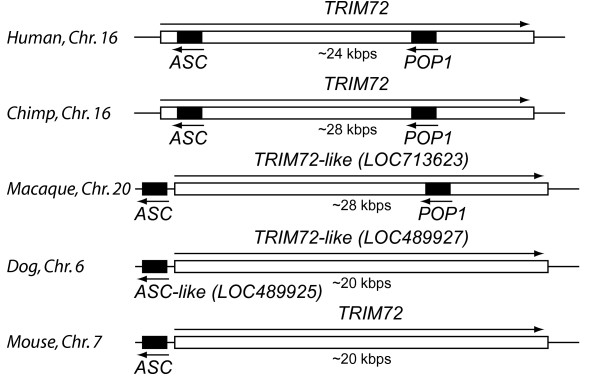
**Genomic organization of ASC and POP1 loci**. For the indicated species, the relationship between the ASC coding region, POP1 coding region (both in black), and the TRIM72 coding region (white box) are shown. Chromosomal (Chr.) assignments and approximate DNA length of the region in kilobasepairs are indicated. Arrows indicate the direction of transcription.

Based on our observation that *POP2 *is absent from the genomes of all domestic mammal species examined, we performed the same screening of the completed genomes of simian non-human primates including *Pan troglodytes *(chimpanzee), *Macaca mulatta *(Rhesus macaque), and with the available whole genome shotgun (WGS) sequences of *Gorilla gorilla *(gorilla), *Pongo abelii *(orangutan; 6 × coverage), and *Callithrix jacchus *(marmoset; 6X coverage). *POP2 *related sequences were found in the genomes of all species examined (except the gorilla genome which is still relatively incomplete). Sequence alignment shows that chimpanzee POP2 is identical to human POP2 at the protein level (Figure 2A). Except for gorilla, the remaining hominid (chimpanzee and orangutan) and Old World (macaque) primate species have a clear *POP2 *ortholog (>90% identity) with open reading frames, but marmosets (New World) do not. No *POP2 *orthologs were detected in the available genomic data from prosimian species. POP1 was also examined with similar results. Maps of the *POP2 *and *POP1 *loci were constructed for those genomes having chromosomal map data (Figure 2B and Figure 3). The genes surrounding *POP2 *and *POP1 *in primate genomes are syntenic and have conserved sequences that have persisted since the emergence of the murine genomes (approximately 80-100 mya). Collectively, these data strongly support the conclusion that POP2 is unique to primates and very likely unique to Old World and hominid primates. As Old World primates are thought to have diverged from New World primates approximately 40 mya, and hominids from Old World primates approximately 25 mya [17], this observation suggests that *POP2 *emerged as a functional gene somewhere between 25 and 40 mya. *POP2 *and *POP1 *thus appear to be among the youngest known human gene products.

### POP2 likely resulted from a contraction of the NLRP2/7 paralog pseudogene CLRX/NOD24

**Figure 4:**
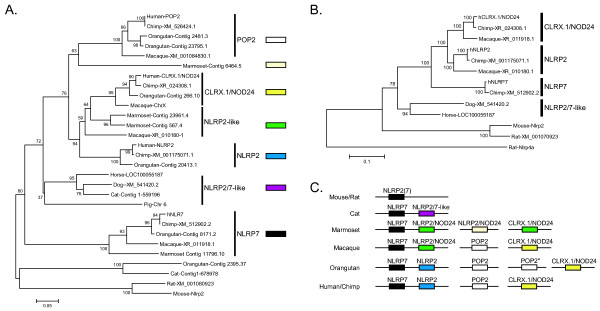
**Duplications in the NLRP2/7 locus and an NLRP2/7 paralog retrogene insertion event presage the emergence of POP2**. A. Phylogenic analysis of POP2-like PYD protein sequences. Colored boxes represent groups of closely related protein sequences based on either grouping with the annotated human sequence (orthologs; black, NLRP7; blue, NLRP2; yellow CLRX/NOD24; or white, POP2) or groups between those identified by homology with their likely orthologs (purple, NLRP2/7; green, NLRP2-like; and beige, POP2-like). Numbers indicate bootstrap values (1000 replicates). B. Phylogenic analysis (maximum likelihood method) of MUSCLE aligned cDNA and predicted full-length NLRP2/7-related nucleotide sequences. The highest log likelihood tree (-14072.9433) is shown (1947 positions). Solid bars indicate grouping of likely orthologs. Numbers indicate bootstrap values. (Note: Macaque ChrX has good homology to CLRX.1 in its Pyrin domain (Figures 2A and 4A) but the full-length Macaque ChrX DNA sequence groups with Dog-XM_541420.2 (bootstrap value 35) and the alignment of the full-length DNA sequence is poor relative to the other genes). C. Graphic representation of NLRP2/7-related genes present in the species examined. Colors correspond with those in A. Marmoset NLRP2/NOD24 (beige) corresponds to contig 6464-5.

Comparison of the identities of POP2-related proteins from all species examined reveals that the number of POP2 PYD-related sequences increases in number during mammalian evolution (one in mouse and four in chimp and human). Phylogenetic analysis of the PYD protein sequences indicates that in the most distant outgroup represented (mouse and rat), the only POP2-related gene is *Nlrp2 *(Figure 4A). In humans, based solely on the PYD protein sequence, the proteins most closely related to POP2 include NLRP2, NLRP7, and the predicted protein product of the CLRX.1/NOD24 locus, suggesting an evolutionary relationship between these proteins. Unlike *NLRP2 *and *NLRP7*, the pseudogene *CLRX.1 *(*NOD24*) [18] contains the stop-codon-rich remnants of its PYD, nucleotide binding domain, and leucine rich repeat coding regions. Like *POP2*, *CLRX.1 or CLRX.1*-like pseudogene sequences are present in all the primate species genomes examined including marmoset (Contig567.4). In our analysis based on PYD protein sequences alone, a number of sequences fail to cluster with either human NLRP7 or NLRP2, suggesting that they represent intermediate forms of the gene (e.g. the NLRP2-like macaque XR 010180.1). To try to resolve whether these sequences represent evolutionary transitions we performed a phylogenetic analysis on the predicted full-length nucleotide and cDNA sequences (Figure 4B). Marmoset and orangutan sequences were excluded as the contigs from these databases were too small to ensure complete coding regions for individual sequences. From this analysis, macaque XR 010180.1 appears to be macaque *NLRP2*. Macaque XR 011918.1 is most similar to *CLRX.1*. Mouse and rat *Nlrp2 *and Dog and Horse *NLRP2/-7*-like are more closely related to human *NLRP7 *than *NLRP2*. This indicates that *Nlrp2 *in mouse and rat is currently a misleading designation. Accordingly, an *Nlrp2/7 *or *Nlrp2/7-*like designation would more accurately reflect its orthologous, syntenic relationship with human *NLRP7*. The presence of both *NLRP2 *and *NLRP7 *in human and non-human primate genomes and the observation that mouse and rat *Nlrp2 *are more closely related to human *NLRP7 *than human *NLRP2 *suggests that an *Nlrp7*-like gene is the common ancestor of the *NLRP7*, *NLRP2*, *CLRX.1/NOD24*, and *POP2 *genes. Given the apparent synteny between mouse *Nlrp2 *and human *NLRP7*, it is highly likely that duplication of the ancestral *NLRP7 *gene (represented here by the common ancestor of mouse Nlrp2 and horse NLRP2/7-like) resulted in the *POP2, NLRP2, and CLRX.1/NOD24 *(Figure 4C). NLRP7 (human and chimp) and NLRP2/7-like (horse and dog) likely represent diversification of the ancestral locus. In the human and chimp genomes, *NLRP2 *is adjacent to *NLRP7 *on chromosome 19. Further, the *CCDC50/Fgf12 *locus which contains *POP2 *on human chromosome 3q23 is not in proximity to other NLRP-related genes suggesting the insertion of the *POP2 *sequence.

### *POP2 *likely originated from a processed pseudogene (retrogene)

The marmoset (*Callthrix jacchus*) genome contains a pseudogene sequence representing a sister clade of POP2 (cjΨ*POP2*). The cjΨ*POP2 *pseudogene resides downstream of marmoset *CCDC50 *and upstream of marmoset *Fgf12 *in same location and orientation as higher primate forms of *POP2*. Further inspection of cjΨ*POP2 *reveals the presence not only of the PYD, but also a nucleotide binding domain, and evidence of leucine-rich repeat coding sequences. Introns between these domains are absent, strongly implicating an insertion of a retrogene copy (processed pseudogene) of one of the NLRs, likely the common ancestor of *CLRX.1/NLRP2*. The ATG start codon is missing and numerous stop codons are present in-frame with the residual PYD sequence. Genes expressed in reproductive tissues are believed to be more likely to generate a heritable processed pseudogene [19] and *POP2 *as well as a number of NLRs are expressed in testis and/or oocytes [12,20-23]. All of these observations are consistent with our interpretation that in marmoset, cjΨ*POP2 *is a processed pseudogene and suggest that in primate evolution, an older, functional NLR retrogene was acquired (likely after the divergence of haplorrhine and strepsirrhine primates), modified, and rapidly selected in Old world and hominid primates to produce *POP2*.

### Macaque POP2 is a functional intermediate between NLRP2 and POP2

**Figure 5:**
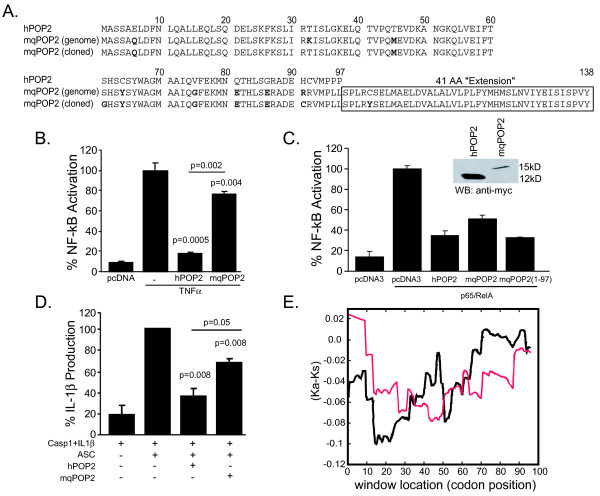
**Macaque POP2 is functionally similar to human/chimp POP2**. Macaque POP2 is functionally similar to human/chimp POP2. A. Comparison of protein sequences from cloned huPOP2 with the macaque genome sequence data and cloned mqPOP2 sequences. Amino acid differences between expected and actual mqPOP2 sequences from two independently isolated clones are shown in bold. Boxed sequence indicates a 41 amino acid "extension" of mqPOP2 absent in human and chimpanzee. B. MqPOP2 weakly inhibits TNFα-mediated NF-κB activation in 293T cells. C. MqPOP2 inhibition of NF-κB p65 is influenced by is 41 AA C-terminal "extension". D. MqPOP2 partially inhibits human inflammasome activity in inflammasome reconstituted 293T cells. E. One dimensional sliding window analysis of Ka and Ks (SWAKK) for human versus macaque POP2 (black) and human versus orangutan (red) (1-97) was performed using the SWAKK algorithm oxytricha.princeton.edu/SWAKK with a window of 30.

Like human and chimp *POP2*, mq*POP2 *is a single exon gene, but unlike *CLRX.1/NOD24*, none have discernable residual coding sequence downstream of their stop codons, suggesting that the loss of NBD and LRR encoding sequences was complete prior to the divergence of these species. In agreement with this data and primate phylogeny [17], mqPOP2 likely retains features in common with the ancestral forms of NLRP2 and POP2 that diverged between approximately 9 and 25 mya. Both the PYD of human NLRP2 and POP2 inhibit NF-κB although their mode of action differs [12,24], but while POP2 has been shown to inhibit inflammasome assembly [12,25], the PYD of NLRP2 does not [24]. To establish whether mqPOP2 is more similar to NLRP2 or POP2 with respect to NF-κB and inflammasome inhibitory properties, we cloned mqPOP2 and compared the inhibitory properties of the protein to that of huPOP2. Comparison of the predicted amino acid sequence from two identical mqPOP2 clones reveals four amino acid substitutions differing from the macaque genome reference sequence (Figure 5A). First, lysine 32 is substituted by threonine, a residue conserved in human and chimp POP2 and in human NLRP2. Residue 61, a serine in the genome database, is a glycine in both clones and appears unique as POP2 (human and chimp), the macaque genome, and the NLRP2 PYD have either a serine or threonine at this position. Position 91 is not conserved between the various POP2 and related sequences, but our clones contain cysteine instead of the expected arginine. MqPOP2 differs from human/chimp POP2 most dramatically by the presence of a 41 amino acid C-terminal extension indicating that this region was likely lost at some point after the divergence of hominid and Old World primates (~25 mya). A cysteine residue at position 102 is a tyrosine in our clones; but this residue occurs in the additional C-terminal 41 amino acids absent in human and chimp POP2. The differences between our clones and the reference sequence for mqPOP2 may represent POP2 polymorphisms or diversification in macaques. Since human and chimp POP2 are identical, purifying selection may have occurred in, or prior to, these lineages. However, a larger number of huPOP2 cDNAs will need to be examined to confirm that huPOP2 is essentially invariant.

Both POP1 and POP2 inhibit NF-κB signaling induced by TNFα. While POP1 inhibits IKKα/IKKβ kinase activity upstream of IκBα phosphorylation and cannot inhibit transactivation by the active p65 subunit [25], POP2 inhibits transactivation by p65 [12]. The isolated PYD of NLRP2 acts similarly to POP1 [24]. Similar to huPOP2, mqPOP2 reduces TNFα-mediated NF-κB activation (Figure 5B). MqPOP2 also inhibits transactivation by NF-κB p65, demonstrating that like huPOP2, mqPOP2 is acting downstream of IκBα phosphorylation at the level of p65. However, the extent of NF-κB inhibition by mqPOP2 is less than that of huPOP2. This may result from the additional C-terminal sequence as its removal (mqPOP2(1-97)) results in a version of the protein with p65 inhibitory activity similar to that of huPOP2 (Figure 5C).

Neither POP1 nor NLRP2 have been demonstrated to prevent inflammasome activation, whereas POP2 is known to block NLR:ASC interaction and inhibits a variety of inflammasomes [12,25]. MqPOP2 is less effective than human at inhibiting inflammasome activation mediated by ASC-overexpression in HEK293 cells (Figure 5D). Although it remains possible that mqPOP2 may more profoundly inhibit other specific inflammasomes or exhibit species specific inflammasome inhibition, these results demonstrate that mqPOP2 possesses the identified functions of its human ortholog.

Given the high degree of similarity to the NLRP2 PYD, mqPOP2 retains more sequence identity with the NLRP2 PYD than with human or chimp POP2 and likely represents a form of POP2 preceding refinements now fixed in the more broadly functional human POP2. The high degree of conservation between human and chimp POP2 suggests that POP2 refined these functions at some point following the divergence of macaques with purifying selection acting at that point to completely conserve POP2 (Figure 5E) prior to the divergence of humans and chimps (~ 6 mya). The divergence of orangutans from other hominid primates occurred around 14 mya [17] and orangutan POP2 is not identical to human and chimp POP2 revealing that as recently as 14 mya, this selection was still acting upon the POP2 locus. The selective pressures driving the appearance of both NLRP2 and POP2 are unknown, however the recent emergence of POP2 suggests a very strong selective pressure impacting reproductive success.

## Conclusions

Of all extant species, chimpanzees and other primates are most similar to humans at the protein and genomic levels. Rodent species however, are widely used as models for biomedical research. Since arrival in the genomic age has accelerated the pace of discovery and increased our knowledge of comparative genomics, the differences between humans and those species used as disease models as well as the need to understand important differences has become increasingly apparent [26]. It has become clear that humans possess greater diversity in protein families involved in inflammation than rodent models used to model inflammatory disease. This is most evident in members of the IL-1/IL-1R family where both agonist and antagonist members are more abundant in humans than mice [27]. Not surprisingly, humans and mice also show differences in the number of NLR proteins potentially involved in inflammasome activity [28]. In this report we have examined the evolutionary history of pyrin-only proteins (POPs) implicated in the regulation of inflammation and find that the emergence of POPs in the mammalian genome is a very recent event occurring roughly at the divergence of Old and New World primates. Further, our evidence suggests that POP2, which can inhibit inflammasome activity, arose from gene duplication events that first gave rise to multiple paralogs of an ancestral *Nlrp2/7*-like gene, followed by diversification of these genes, retrogene insertion of an *NLRP2*-like paralog, and rapid loss of the signature NBD and LRR encoding regions to yield a functional POP2 gene. MqPOP2 approximates the most distant intact ancestor of POP2, predating the divergence of humans and chimps by approximately 5-10 million years, and possesses both the NF-κB p65 and inflammasome inhibitory properties reported for the human protein. Collectively, these data suggest a strong selective pressure driving the recent emergence of a small Pyrin-only protein inhibiting both NF-κB signaling and the activation of multiple inflammasomes that corresponds with the emergence of hominid and Old World primates.

The marmoset genome clearly reveals the prior insertion of a retrogene copy of an *NLRP2*-like transcript at the developing *POP2 *locus. By the emergence Old World primates, traces of the non-PYD coding portions of the retrogene are no longer apparent. In humans, a functional polyadenylation sequence is present within the 3' UTR of POP2 [12]. Similar sequences are present in the 3' UTR of chimp and mqPOP2. Beyond reflecting the selective pressures acting on the POP2 gene, these features lend additional support to the emerging hypothesis that retrogenes are frequently functional, add to the complexity of the genome and may confer important regulatory functions as recently demonstrated for fibroblast growth factor 4 [29].

Do the recent emergence of POP2 and other inflammatory regulators (e.g. POP1, COP, and INCA) reflect an increased need to control inflammation during the most recent stages of primate evolution? This is a difficult question requiring further investigation. However, considering that a strong selective pressure likely drove the evolutionary development of POP2, it is reasonable that POP2 might act by offsetting inflammatory events that decrease reproductive success. Inflammation of the reproductive organs would be a direct example. Recently, mutations in NLRP14 (although not yet demonstrated to initiate an inflammasome) have been implicated in failed spermatogenesis and may dysregulate inflammation or promote apoptosis [21,22]. Interestingly, although inducible in monocytic cells, POP2 is expressed constitutively in the testis [12] and could thus potentially have a role in modulating NLRP14 function. As a more extreme example, NOMID, one of the most severe autoinflammatory diseases, is one outcome of mutations in NLRP3, an inflammasome-initiating protein modulated by POP2 [12,25]. Approximately 20% of individuals with NOMID die before adulthood. Identification of mutations or deletions in POP2 correlating with male reproductive system failure would lend support to this idea. The involvement of NLRP2 and NLRP7 in inflammasome inhibition [30] and a connection between their presence among maternal RNAs in oocytes and the formation of hydatiform moles [31-33], a form of reproductive failure, further suggest that some NLRs may have roles in both inflammation and reproductive success. Nevertheless, as no specific disease associations have been identified for the *POP2 *locus, these ideas remain speculative and await the results of further studies exploring the role of POP2.

In summary, the recent emergence of the highly selected and functional POP2 gene in higher primates, apes, and humans suggests a strong selective pressure among these species for the functions of the POP2 protein. Although our understanding of the biological role of POP2 is in its infancy, its apparent biochemical roles in regulating NF-κB activity and inflammasome formation suggest a variety of possibilities that may shed light on important differences between higher primates and other mammalian species.

## Methods

### Database search strategy, and sequence prediction

TBLASTN searches of the NCBI genome databases for human (build 36.3, March 2008), chimp (Build 2.1, October 2006), macaque (Build 1.1 June 2006), dog (build 2.1, September 2005), cow (Btau_4.0, August 2008), chicken (build 2.1, November 2006), and mouse (build 37.1, July 2007); Washington University genome databases for orangutan (*Pongo pygmaeus abelii *2.0.2 contigs) and marmoset (*Callithrix jacchus *2.0.2 contigs) were performed without the low complexity filter and with default expect (E) values using the huPOP2 (PYDC2, AY858112.1) as the query. Subsequent analysis of the marmoset genome used the Ensembl database (*Callithrix jacchus *3.2). TBLASTN searches of the nr/nr and non-human, non-mouse EST databases were also performed as above.

### Phylogenetic analysis of nucleotide and protein sequence

Predicted or known nucleotide and protein sequences for all the identified loci were aligned with each other using CLUSTALX [34] or MUSCLE [35]. Phylogenetic trees were constructed using the MEGA 3.1 [36] or MEGA 4 [37] software packages. For nucleotide alignments trees were constructed based on the Maximum Likelihood method (Tamura-Nei model [38]) with gaps and missing data eliminated and bootstrapped with 1000 repetitions. Protein alignments were analyzed and neighbor-joining trees were constructed based on the amino acid: number of differences algorithm with pairwise deletion of gaps and bootstrapped with 1000 repetitions.

### Cells

The kidney epithelial fibroblast cell line HEK293T was cultured in DMEM with 10% FBS, 1% L-glutamine and 0.1% penicillin/streptomycin cocktail at 37°C, 5% CO_2_. Peripheral blood leukocytes from Rhesus macaque were the kind gift of Drs. Deborah H. Fuller and Michael Murphey Corb.

### RNA isolation and cloning

RNA was isolated from Rhesus macaque peripheral blood leukocytes using RNEasy (Qiagen) reagents. RNA was treated with DNAaseI and the macaque POP2 specific primers 5'-AA GAATTC ATG GCA TCT TCT GCA CAG CTG G-3' and 5'-AA CTCGAG TCA ATA TAC TGG TGA TAT AGA TAT TTC-3' were used with the One-Step RT-PCR kit (Qiagen) to amplify mqPOP2 cDNA. The cDNA product was digested with EcoRI and XhoI and ligated into pcDNA3 (Invitrogen). Two independently isolated clones were sequenced (Genewiz) and returned identical nucleotide sequences. The sequence of this clone of macaque POP2 has been deposited at GenBank (Accession Number: JF327668).

### NF-κB reporter assays

NF-κB Luciferase reporter assays were performed as previously described [12]. Briefly, HEK293T (2 × 10^5 ^cells/well) were seeded in 6-well plates, transfected with 100 ng 3 × NFκB-luciferase reporter and 50 ng NFκB-p65 with 1 μg myc-huPOP2, myc-macaque POP2, or empty vector using Fugene6 (Roche) as described by the manufacturer (2.5:1 ratio Fugene6:ug DNA. Cells were harvested 18 hours post-transfection, lysed in 1 × reporter lysis buffer (Promega), and luciferase activity was assayed using a Victor3V luminometer (Perkin-Elmer). For TNFα-induced NFκB activation, cells were stimulated with TNFα (10 ng/ml) 3 hrs prior to harvesting. All relative light unit values were normalized to total protein as described [39].

### Inflammasome inhibition assays

HEK293T cells were seeded (5 × 10^4 ^cells/well) in 24 well plate a day before the experiment. For inflammasome reconstitution, cells were transfected with plasmids encoding Pro-caspase1 (50 ng), Pro-IL1β (200 ng), and ASC (400 ng) in presence or absence of POP1, full-length huPOP2 or macaque POP2 (500 ng). At 18 hrs post-transfection, culture supernatants were harvested, centrifuged briefly to remove any cellular debris and immediately used for the measurement of secreted IL-1β by human IL-1β ELISA kit (eBiosciences) as per manufacturer's instructions or stored at -20°C for later use. For ASC over-expression, similar experiments were performed by using 400 ng of ASC instead of 20 ng. Note that in ASC over-expression experiments there was no transfection of any NLR.

### Immunoblotting

HEK293T cells were transfected with 1 μg of myc-tagged huPOP2, mqPOP2 WT or mqPOP2 1-97. Cells were lysed 24 hr post-transfection using 1% NP-40 lysis buffer [50 mM Tris-HCl (pH 7.4), 150 mM NaCl, 1% NP-40 (v/v), 2 mM EDTA, 2 mM DTT with protease inhibitors (Roche)], run on SDS-PAGE (Bio-Rad) and transferred to PVDF membrane. After blocking with 3% milk/PBS, membrane was probed using mouse anti-myc antibody (1:1000, Millipore) overnight, followed by HRP-conjugated goat anti-mouse antibody (1:5000, Sigma) for 1 hr. Protein bands were detected using SuperSignal West Pico detection reagents (Thermoscientific).

### Statistical analysis

Experiments were repeated at least three times unless indicated otherwise. Statistical significance between experimental groups were measured by Student's *t-test *with p < 0.05 considered significant.

## List of abbreviations

PRR: pattern recognition receptor; TLR: Toll-like receptor; NLR: nucleotide-binding, leucine repeat receptor; PYD: pyrin domain; CARD: caspase recruitment domain; COP: CARD-only protein; POP: pyrin-only protein; cjΨPOP2: *C. jacchus *POP2 pseudogene.

## Competing interests

The authors declare that they have no competing interests.

## Authors' contributions

MA isolated and cloned macaque POP2, performed functional assays, analyzed data, and assisted with manuscript preparation. TF conducted BLAST searches. JH conducted BLAST searches, made alignments and phylogenetic trees, made the figures, analyzed the genomic contexts, analyzed data, and wrote the text. All authors read and approved the final manuscript.
